# Correlates of Tobacco Use Among People with Mental Illness Within Asia: A Scoping Review

**DOI:** 10.1007/s10597-024-01336-w

**Published:** 2024-08-10

**Authors:** Parul Parul, Bindu Joseph, Sunil Datta, Muhammad Aziz Rahman

**Affiliations:** 1 Present Address: Institute of Health and Wellbeing, Federation University Australia, Berwick, VIC Australia; 2 School of Nursing, Institute of Health & Management, Melbourne, Australia; 3 The Royal Melbourne Hospital, Melbourne, Australia; 4 Collaborative Evaluation and Research Centre (CERC), Federation University Australia, Churchill, Australia; 5 Faculty of Public Health, Universitas Airlangga, Surabaya, Indonesia

**Keywords:** Tobacco, Smoking, Mental illness, Prevalence, Predictors, Asia

## Abstract

Tobacco use among people with mental illness is one of the contributing risk factors for premature morbidity and mortality. Two in three people with mental illness are current smokers with deteriorating physical health and quality of life. This review outlines the prevalence and predictors of tobacco use among people with mental illness in Asia. Twenty-five cross-sectional studies were selected from the exhaustive search of databases. Ten countries emerged based on the number of studies conducted within Asia among people diagnosed with mental illness and tobacco use, namely, India (8), China (7), Pakistan (2), Singapore (2), Sri Lanka (01), Japan (01), Jordan (01), Malaysia (01), Korea (01), and Taiwan (01). The prevalence of tobacco use was in the range of 3.6% to 89.4%, with the measure of precision at 95% confidence. The highest and lowest prevalence was reported in China, followed by India. Being male, separated, lower education, unemployed, lack of family support, a psychotic diagnosis, lack of knowledge, motivation, and a coping mechanism were predictors of tobacco use. This review emphasized the extent and predictors of tobacco use among this vulnerable group that need to be reflected while initiating and implementing cessation strategies by healthcare providers.

**Supplementary Information:**

The online version contains supplementary material available at 10.1007/s10597-024-01336-w.

## Introduction

Mental illness is a global health concern that imposes a significant burden on individuals and society (Thyloth et al., 2016). People with mental illness usually face hindrances in adopting healthy behavioral habits, eventually making them most vulnerable to deteriorating activities such as tobacco consumption, overeating, or aggressive behaviors. Ignoring these activities results in long-term consequences on mental health (Romain et al., 2020). Globally, tobacco alone is killing more than 8 million people in a year and emerging as an epidemic in public health (World Health Organization [WHO], 2021). Thirteen percent of the global disease burden is related to mental illness, substance abuse, outstanding cardiovascular disorders, and cancer (WHO, 2018). Though a significant decline in tobacco was evident in the general population through policies and the Framework Convention on Tobacco Control (WHO, 2022), the picture is still not the same for people with mental illness.

The prevalence of tobacco use among people with mental illness is high globally (De Leon & Diaz, 2005). Developed countries like the USA (Prochaska et al., 2017; Smith et al., 2014), the United Kingdom (Peckham et al., 2021; Szatkowski & McNeill, 2015), Canada (Dahal et al., 2020; Gerber et al., 2003; Margolese et al., 2004) and Australia (Lawrence et al., 2013; Mendelsohn et al., 2015) provided evidence of a high prevalence of tobacco use among people with mental illness, but data is scarce in the Asian context. Furthermore, the margins of tobacco prevalence in the Western world declined in recent years, whereas the Asian region still has the highest rates of tobacco use (WHO, 2021). Additionally, Asia is one of tobacco's largest producers and consumers, and around one-third of total tobacco use is concentrated in the Southeast Asia region (WHO, 2015). It is estimated that by 2025, the prevalence of tobacco will track down to 25.1%, which is still the highest among all regions globally (WHO, 2021). There are several factors (biological, social, and economic) that contribute to the continuation of smoking behavior, where smoking either becomes a coping strategy or a rationale for suppressing the side effects of psychotropic drugs (Fernando et al., 2019).

Tobacco use has a rewarding effect on mood and cognition for a short time, but continual usage leads to dependence and later withdrawal symptoms such as low mood, irritability, poor concentration, restlessness, and anxiety (Kutlu et al., 2015; Taylor et al., 2021). Moreover, evidence suggests that people with mental illness are more likely to continue smoking and are less likely to quit (Pal & Balhara, 2016). No single unifying hypothesis explains the relationship between tobacco and mental illness, though the self-medication hypothesis is widespread in clinical settings, claiming that smoking relieves the side effects of psychotropic drugs (Sharma et al., 2016). Consequently, smoking-related physical illness shortens the life span (10–25 years) among people with mental illness compared to the general population (WHO, 2020). Surprisingly, rare evidence suggests that smokers with mental illness genuinely express a desire/interest in quitting (Taylor et al., 2021), which ultimately highlights the importance of screening for tobacco use at the initial stages of the management plan (Khobragade et al., 2020; Ward & Drus, 2019).

Elucidating predictors of tobacco use among people with mental illness is a multifactorial process influenced by various contextual factors, that is, social, environmental, psychological, and genetic factors (Minichino et al., 2013). Latest findings from the Western world confirmed that males, young age, lower education status, co-dependency, psychotic diagnosis, and rural residence are predominant determinants of tobacco use among people with mental health issues (Okoli et al., 2019). Additionally, predictors like peer influence, parental history of smoking, and social media influence and availability in different forms contribute to continued usage (Thakur & Paika, 2018). Smoking contributes to disproportionate health issues and enhances financial burdens that eventually hamper the overall quality of life (Forman–Hoffman et al., 2016). Inadequate support for people with mental illness, along with stigma and discrimination, hampers the initiation and access to quality care (Abdisa et al., 2020; Knaak et al., 2017). Understanding these predictors is paramount for providing cessation support for people with mental illness.

Some of the most effective strategies to control tobacco usage include the comprehensive implementation of health policies, increased taxes, awareness in the media, and campaigns against tobacco consumption. Moreover, discontinuing tobacco use not only defeats psychiatric symptoms but also lowers the risk of heart disease, stroke, and cancer, improving mental health outcomes (National Center for Health Statistics, 2017). There are no explicit guidelines for such vulnerable groups to restrict the usage of such products (Mukherjee & Awasthi, 2022). Understanding the extent and impact of clinical and non-clinical factors of tobacco use among people with mental illness will aid in curbing such deteriorating comorbidity, which impacts overall health and well-being. In- addition such data will assist in improving tobacco cessation services for people with mental illness. Therefore, the current scoping review aims to explore the extent and predictors of tobacco use among people with mental illness for a comprehensive understanding that ultimately provides a concrete framework for executing effective cessation in Asian settings.

## Methods

### Eligibility Criteria

The present scoping review was executed based on Arksey and O'Malley's adopted methodological framework (Arksey and O’Malley, 2005). This review follows PRISMA-ScR (Preferred Reporting Items for Systematic Reviews and Meta-Analyses Extension for Scoping Reviews) guidelines (Tricco et al., 2018) and updated methodology (Levac et al., 2010). Primary studies published in peer-reviewed journals until June 2022 involved adult participants with mental illness who were consuming tobacco in Asian countries (Table 1). The key definitions of the present review are (i) "*tobacco use refers to smoking and smokeless tobacco products in the varied form" and (ii) "people with mental illness refers to people being clinically diagnosed with mental disorders that interfere with individual’s cognitive, emotional or social abilities as per ICD or DSM classification"(APA. *2022*).* The inclusion criteria for this review followed the "population, concept, and context" format (PCC), which is adapted to facilitate a broader search and ensures different methodologies (Peters et al., 2015). The quality of the selected papers was appraised through the Critical Appraisal Skills Program (CASP, 2022). Table 1 presents the detailed inclusion and exclusion criteria considered in the present review work.

**Table 1:** Inclusion and exclusion criteria

Criteria	Inclusion	Exclusion
Population	Tobacco use among people (aged ≥ 18 years) diagnosed with mental illness	People using other substances after being diagnosed with mental illness
Concept	Prevalence and predictors of tobacco use among people with mental illness	Association or relationship between tobacco use and mental illness
Context	Asia	Countries in Africa, Europe, North and South America and Australia/Oceania continents
Design	Primary studies: descriptive, comparative, cross-sectional, cohort, case–control & quasi-experimental studies	Review papers, editorial letters, reports, and conference abstracts
Language	English	Language other than English

### Information Sources and Search Strategy

An extensive search of published articles from databases with truncation and proximity operators was executed from inception to June 2022. Six distinguished databases were searched: CINHAL, MEDLINE, PsycINFO, Web of Science (WoS), SCOPUS, and PubMed. Furthermore, reference lists of all qualified primary studies were explored to identify any missed articles in databases. Initially, the search was executed in a few research publication databases to analyse text words for tobacco use and people with mental illness in the title and abstract; later, index terms were explored and implemented in each database. Search terms were tobacco OR nicotine OR smoking OR "cigarette*" OR chewing OR bidis OR khaini OR gutkha OR betel OR zarda OR hookah OR "smok*"OR "smokeless*" OR "SLT*" AND Mental* (illness* OR health OR disorder* OR issues* OR severe* OR common*) OR CMI* OR SMI*OR psychosis*OR psychiatric* OR schizophreni* OR BPAD, OR bipolar* OR depression* OR depressive* OR mania* OR anxiety* AND Asia* along with names of each country. The final search strategy has been developed in consultation with our institutional librarian and discussed with all co-authors*.*

### Selection of Sources and Data Charting Process

Adhering to search strategies, one author (PP) exported all articles (n = 8285) into Endnote for duplicate removal and later imported all articles into Covidence. The initial screening was conducted by two authors (PP and BJ), followed by abstract screening which was carried out independently by another two authors (PP and MAR), adhering to predetermined inclusion criteria. The next step was the full-text screening of eligible articles, which was independently executed by PP and BJ, with votes moderated by MAR for the final decision. In the end, conflicts were resolved through discussion among team members, along with a final decision by the fourth author (SD). The data was segregated after a thorough discussion and trailed by a JBI-approved PICO tool guide with approval from all reviewers. Data extraction includes the author's name (s), year of publication, name of the country, sample size, clinical diagnosis, setting, tobacco screening tool, design, prevalence, and predictors of tobacco use.

### Quality of Selected Studies

Only peer-reviewed articles were selected in the final search maintaining scientific standards in content. Two authors independently read all full-text articles while including them in a final search based on predefined criteria. Any disagreement/doubt was resolved with the rest of the team members. Moreover, the quality of the selected papers was appraised through the Critical Appraisal Skills Program (CASP, 2022). The CASP tool uses a distinct set of comprehension questions for each specific form of research method (Quality Assessment Tool for Observational Cohort and Cross-Sectional Studies). The headings under this checklist were clear aims, methodology, design, recruitment, data collection, ethical considerations, data analysis, findings, and research value. Cumulative scores from each subheading for every article were categorized as A, B, C, or D. All twenty-five articles fall into categories of either A, B, or C (refer to the supplementary Table 3 for further details).

## Results

### Characteristics of Sources of Evidence

**Fig. 1:**
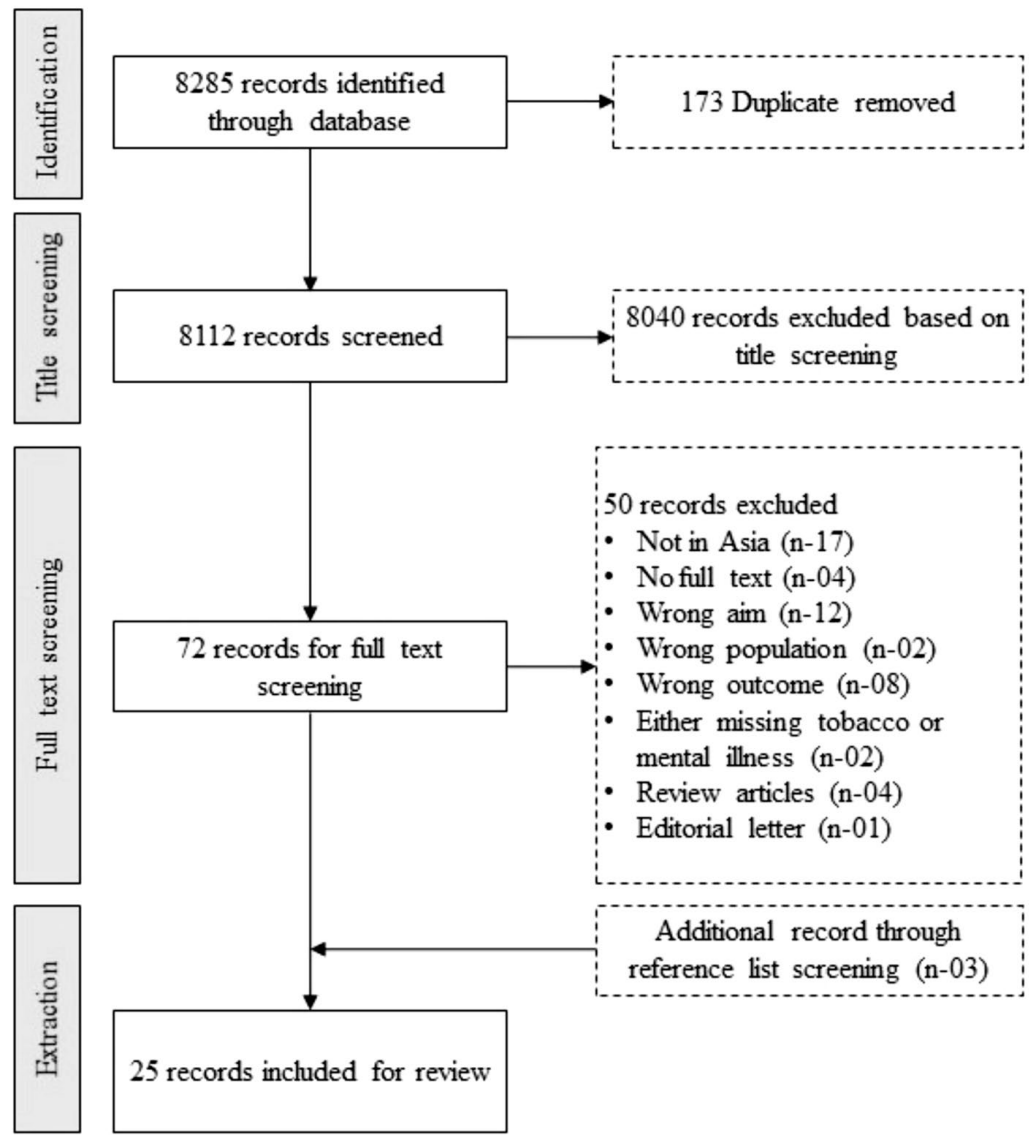
PRISMA flow diagram in the present study

**Fig. 2:**
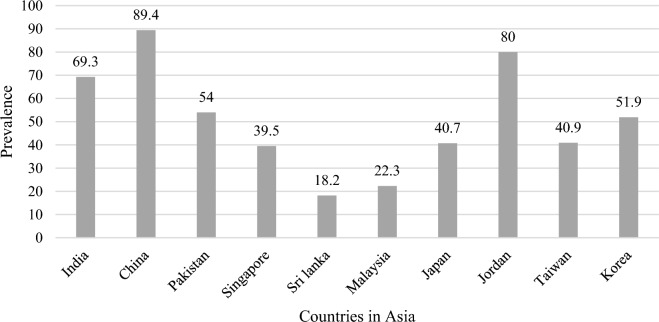
Country-wise highest prevalence of current tobacco use in Asia

The initial search yielded 8285 articles (refer to Fig. 1); twenty-five articles met the inclusion criteria and were included for a final review. All studies had cross-sectional designs. Figure 2 presents the highest prevalence among selected countries from Asia. Considering the heterogeneity among the ten countries, India (n = 8) and China (n = 7) have the highest number of articles with broader coverage of tobacco use prevalence and factors among people with mental illness. Most studies were conducted in tertiary care hospitals (n = 23), and only two were in community settings. All the participants were stable, non-aggressive, and cognitively capable of giving consent for data collection. Fagerstrom test for nicotine dependence (FTND) was used to screen tobacco dependence (n = 13), while four records did not mention a tool (n = 4) for tobacco screening, and few mentioned the self-report method (n = 8) as a screening tool, which assuredly brings chances of recall biases. Surprisingly, only two studies used carbon monoxide (CO) in an expired breath to validate self-report status (Shinozaki et al., 2011; Zhang et al., 2010).

### Summary of Evidence

#### Prevalence of Tobacco Use

As per Fig. 2, the selected studies show the highest prevalence of current smoking, with overall ranging from 3.6% to 85%. Most studies (n = 17) included both genders, though five had male participants only (prevalence range of 25.3% to 80%), and only three had female participants (prevalence range of 3.6% to 47.1%). The mean number of cigarettes smoked daily was 20 to 30 (Aziz et al., 2017; Chong & Choo., 1996; Shinozaki et al., 2011). Only one record reported more than 51 daily cigarette consumption (Ranganathan, 2017). Moderate nicotine dependence based on scores from the FTND scale was revealed amongst patients with mental illness in most studies (Aziz et al., 2017; Vatss et al., 2012), whereas one study reported a high level of nicotine dependence (Swaroop et al., 2014). Moreover, evidence from extracted studies showed that smoking was the most prevalent form of tobacco use in Asia. Only two studies discussed smokeless tobacco (Batki et al., 2009; Vatss et al., 2012). All the participants were diagnosed with severe mental illness (SMI) as the distribution based on the clinical diagnosis, which was either schizophrenia (n = 11), combined schizophrenic and other psychotic disorders (n = 11), depression (n = 02), and bipolar disorders (n = 1). A detailed description of the prevalence and predictors of included studies is available in supplementary tables 1 and 2.

#### Predictors of tobacco use

##### Socio-demographic predictors

Age: The mean age of smokers with mental illness was 39.9 ± 14.3 and 30.6 ± 10.49 years in males and females respectively. Smoking prevalence was higher after 20 years of age (Zhang et al., 2010), whereas another study evident that the prevalence declined after the age of 55 years (Ma et al., 2009).

Gender: Smoking was the most prevalent form among males ranging from 18.2% to 96%, where the cigarette was the most readily available form of tobacco. Among females, smokeless tobacco (SLT) was the most widespread (Swaroop et al., 2014). Only two studies explored smokeless tobacco use among males (Srinivasan & Thara, 2002; Vatss et al., 2012). Furthermore, solitary female participants with smoked tobacco were in two studies where the prevalence was low, with a range of 3.6% to 12.14% (He et al., 2014; Khobragade et al., 2020) as evidence supported that sociocultural factors and norms confined females from smoking, especially in Asian countries.

Education: Most participants were illiterate or had only education till primary. Only one study showed minimal smoking among participants beyond secondary or higher education (Wang et al., 2015). Moreover, higher nicotine dependence was associated with fewer years of education associated with their smoking habit (Kim et al., 2013).

Marital status: Being single or separated amplified their likelihood of smoking due to a lack of support from their spouse or children. As a bachelor or separated they were more vulnerable to shame due to cultural expectations of marriage, eventually leading to isolation and continual smoking (He et al., 2014). In contrast, complex personal relationships, social stress, and societal pressure influenced them towards smoking (Ma et al., 2009). However, one study found no association between marital status and smoking (Shinozaki et al., 2011).

Family history and available support: The influence of family sanctioning on tobacco use was one of the sociocultural factors impacting smoking (Srinivasan & Thara, 2002). Most participants depended on their families to buy tobacco products (Vatss et al., 2012), while few family units attempted to restrain smoking under social, cultural, and family influences (Srinivasan & Thara, 2002). Family support was a decisive factor in tobacco use initiation and continuation (n = 10) in terms of a positive family history of smoking or getting financial support to buy tobacco products.

Employment: The unemployment range among people with mental illness was 38% to 91% (Wang et al., 2015; He et al., 2014). This cohort experiences a high level of unemployment due to disturbance in cognitive and social impairment. Simultaneously, being employed facilitated access to tobacco, which was a factor for continued smoking (Ranganathan, 2017; Swaroop et al., 2014). Moreover, sharing tobacco in the workplace is a common and acceptable trend (Kar et al., 2020). Tobacco access was one of the imperative non-clinical factors for predicting smoking behaviour (Srinivasan & Thara, 2002).

Socioeconomic status (SES): Socioeconomic status and family systems in Asian countries substantially influence smoking behaviour (He et al., 2014). Residing in rural areas and having a lower socioeconomic status leads to stress, ultimately provoking smoking (Ranganathan, 2017). On the contrary, two studies reported that social class was not associated with current smoking (Shinozaki et al., 2011; Srinivasan & Thara, 2002).

##### Clinical Predictors

To relieve symptoms of mental illness: People with mental illness are inclined towards tobacco use, as manifested by fewer positive symptoms in schizophrenia (Zhang et al., 2007), which is popularly known as the Self-medication hypothesis, where substances like tobacco reduce psychiatric symptoms by acting as a coping mechanism to deal with underlying mental health issues (Fang et al., 2019). In contrast, some studies reported that smokers diagnosed with psychotic symptoms experience exceptionally severe positive symptoms (Liao et al., 2002; Vatss et al., 2012; Zhang et al., 2007) caused by free radicals and inflammatory responses (oxidative stress) from substances. Tobacco users suffered more severe positive symptoms (Fang et al., 2019; He et al., 2014) as evidenced by higher scores on Brief Psychiatric Rating Scale (BPRS) (Chong & Choo, 1996), Scale Assessment Positive Symptoms (SAPP) (Vatss et al., 2012) & Positive and negative symptoms of schizophrenia (PANSS) scales (Liao et al., 2002; Vatss et al., 2012); Zhang et al., 2007) another study shows no effect of smoking in terms of scores of BPRS (Liao et al., 2002) while other two studies found an inverse association between tobacco use and the therapeutic efficiency of psychotropic drugs (Ranganathan, 2017; Srinivasan & Thara, 2002). Moreover, usage of tobacco leads to non-adherence to psychotropic medication with less than 50% of tobacco users following treatment as compared to 75% of non-tobacco users (Kar et al., 2020). Also, people with mental illness fear withdrawal symptoms from ongoing conditions (Asharani et al., 2020).

Diagnoses: All studies confirmed participants' diagnoses through the International Classification of Diseases (ICD) or DSM (Diagnostic and Statistical Manual of Mental Disorders) classification. Half of the studies were on people with a diagnosis of schizophrenia (n = 12), followed by severe mental illness (n = 8), depression (n = 2), and bipolar disorders (n = 1). Two studies were on psychiatric illnesses where the specific diagnosis was not mentioned.

##### Individual Predictors

Lack of knowledge: A lack of knowledge about the harmful effects of tobacco use was apparent among people with mental illnesses (Wang et al., 2015). Only one record with merely 12% of tobacco users perceived that tobacco worsened their psychiatric symptoms (Srinivasan & Thara, 2002). Participants were not even aware of the services available in the hospital (Asharani et al., 2020; Khobragade et al., 2020). Additionally, scarcity of data was evident regarding the screening of tobacco usage (Kim et al., 2013; Wang et al., 2015). Surprisingly, 90.7% of smokers denied that tobacco use contributed to their illness (Ranganathan, 2017).

Lack of motivation: People with mental illness lack the motivation to quit smoking as they consider it a part of their habit, and participants enjoy consuming it (Asharani et al., 2020; Aziz et al., 2017; Kim et al., 2013; Ma et al., 2009; Wang et al., 2015). Furthermore, participants firmly believed tobacco use as a substitute for other harmful substances (Hapangama et al., 2013). Reluctance to abstain from tobacco use was evident with the justification that smoking does not affect physical health (Asharani et al., 2020; Fang et al., 2019; Li et al., 2017; Srinivasan & Thara, 2002).

Smoking as a coping mechanism: Tension-relieving effects (Srinivasan & Thara, 2002) and resolving anxiety (Aziz et al., 2017; Ranganathan, 2017) were the most prominent factors for the continued use of tobacco. Moreover, studies reported that tobacco acts as a stress-relieving method, fading the impact of psychiatric symptoms (Wang et al., 2015; He et al., 2014). Another study also supported that tobacco usage improved accuracy and efficiency under attention domains (Asharani et al., 2020; Hapangama et al., 2013).

## Discussion

This scoping review explored the prevalence and predictors of tobacco use among people with mental illness. It indicated the persistently high prevalence of tobacco use among people with mental illness, consistent with a meta-analysis of a global review across different geographical regions (highest in Asia and lowest in South America) (Fornaro et al., 2022). In addition, recent works in the Western world (Fornaro et al., 2022; Okoli et al., 2019) confirm the extent and predictors of tobacco use in this vulnerable group, but literature is scarce in Asian settings. Continuing to this evidence, the pattern of daily smoking prevalence was highest in the Southeast Asian and European regions, which eventually constituted 45.7% of daily smokers, with participants mostly from India, China, and Indonesia (Peacock et al., 2018).

All studies in this review adhere to a cross-sectional design aligned with recent global reviews (Fornaro et al., 2022). Most studies were undertaken in tertiary care hospitals since inpatient and outpatient services had been identified as the best settings to screen tobacco use (Pipe et al., 2022). However, the research conducted in community settings appeared promising because of the adequate sample size to generalize the findings at the grassroots level (Fornaro et al., 2022). This review also indicates the need for standardized tobacco screening tools, as discrepancies in the categories were evident based on different sources and cultural settings. Descriptions of tobacco usage are noticeable (Global Adult Tobacco Survey Collaborative Group, 2020), as not all records considered it while collecting data. Furthermore, the duration of smoking and the number of cigarettes smoked were also not comparable in most of the studies, as some interpreted their result based on 100 cigarettes in their lifetime, while others conveyed one cigarette in a day (Asharani et al., 2020; Li et al., 2017).

Smoking is one of the most common forms among males in Asia, which is analogous to the findings from the Western world (Okoli et al., 2019; Prochaska et al., 2017). Financial independence and sociocultural acceptance of smoking among males in Asian settings contribute to continued tobacco use, which is generally perceived as symbolizing masculinity and the socializing nature of males. Likewise, there is often less stigma attached to smoking in Asian societies compared to females. Females had a lower rate of smoking being diagnosed with mental illness (Rahman et al., 2015), as they are considered primary caregivers within their families. Additionally, women generally face stronger social stigma and rejection for smoking, reflecting their accountability for health-promoting behaviors.

A qualitative study in the Western world supports the scoping review findings, as a lower level of education and being single or separated corresponds to tobacco use (Keller-Hamilton et al., 2019). This is manifested as lower education leading to lower access to health information and reduced access to preventive services. Likewise, single or separated individuals experience poor social support, which may contribute to higher chances of indulging in tobacco use. Individuals with a family history of tobacco use have a higher chance of smoking (Kelkar et al., 2020; Kar et al., 2020). If parents or siblings are smokers, it enhances the acceptability and availability of tobacco in the house, which serves as a strong factor for the initiation and continual smoking. Another significant factor is the comorbidity of psychotic disorders, which is strongly associated with tobacco use (Brar et al., 2021; Fornaro et al., 2022; Prochaska et al., 2017), as evident in the present scoping review. This comorbidity is linked to varied interrelated factors such as self-medication and social stigma associated with mental illness. The current scoping review highlighted that the high nicotine level is related to alleviating psychiatric symptoms (self-medication). Also, persistent stress, when diagnosed with psychotic illness, leads to social stigma and isolation, which provokes higher smoking as a coping mechanism.

Lack of knowledge regarding the consequences of smoking on physical health in this vulnerable group was evident necessitating the need for healthcare providers to adhere to World Health Organization guidelines for managing physical health among adults with mental illness (WHO, 2018). Another attribute of the predictors was the lack of motivation to quit smoking, and the comparison in motivation to quit smoking among people with mental illness, and the general population showed that people with mental illness were the least motivated to quit smoking (Siru et al., 2009) which was evident in the present scoping review among participants through statements such as the use of tobacco for pleasure or as part of their habit (Bartlett et al., 2016). This raises the need to explore further factors assessing motives for smoking among people with mental illness (Schermitzler et al., 2021), as it is a critical predictor of successful cessation interventions (Manolios et al., 2021).

There is substantial evidence of concern as only two records indicate screening of tobacco use by healthcare providers (Kim et al., 2013; Wang et al., 2015) which emerges as a significant barrier to abstaining from tobacco (Manolios et al., 2021). There is a low rate of healthcare providers employing brief strategies (ask-advice-refer) to achieve desired health outcomes (Meijer et al., 2019) even recent findings from the Western world (Siddiqi et al., 2022) confirmed that competing diagnoses, the belief that patients will be non-adherence to cessation treatment, lack of training among healthcare professionals and poor organizational support were some prominent barriers that emerged while planning cessation support services. Smoking cessation must be carefully integrated with the treatment plan, as mental illness is not a contraindication to pausing smoking (RACGP, 2011). A scarcity of precise data regarding tobacco use among people with mental illness, especially in the Asian context, warrants a deeper understanding for further research and public health attention. Stakeholders like healthcare providers, policymakers, and researchers need continued efforts to address this significant issue, which has a damaging impact on health and society (Prochaska et al., 2017).

## Limitations

The limitation of this scoping review was that only twenty-five pieces of research evidence came up in the selected searches on databases in the Asian context, which might not be concisely specified in terms of the name of diagnosis within mental illness, which eventually makes it difficult to say it as representative of the general population of people with mental illness in Asia. Studies published in English were only included. There is also a discrepancy in smoking categories as in most of the studies, smoking prevalence was assessed for current smokers, with not much emphasis on lifetime or former smokers. The authors intended to categorize data into subgroups based on diagnoses but were unable to do so due to limited availability for the same. To generalize the results, authors sought other clinically relevant variables, such as treatment variables and the severity of symptoms, which were also unavailable to comprehend.

## Conclusion and Future Directions

This is the first review in our knowledge covering the broad availability of evidence for tobacco use among people with mental illness in Asia. The high prevalence of tobacco use concurrent with mental illness, especially in Asian men with psychotic diagnoses, is a prime concern, as evident through the present scoping review. Exploring the connection of socio-demographic, clinical, and personal factors aids in understanding the broad picture, which subsequently aids in the formulation of tailored interventions for a successful outcome in terms of low smoking rate among people with mental illness in Asia. The review concluded that the prevalence rate in Asia is high compared to the rest of the regions globally owing to varied demographic and cultural factors where smoking is more acceptable. Another crucial factor in the Asian context is the lower level of education and awareness, triggering health risks correlated with tobacco. Understanding the individual factors (knowledge, motivation, and a coping mechanism) plays a vital role in abstinence from tobacco.

In terms of future guidance, understanding the extent and factors of tobacco use will inform targeted public health interventions by addressing specific needs and challenges among people with mental illness in Asia. The findings will also guide healthcare providers to offer comprehensive support addressing mental health issues and smoking cessation simultaneously. In addition, the results of this study will guide the development of policies and future research to support treatment strategies within mental health services located in Asia.

## Implications

This review paper highlights the need to recognize and consider the predictors of tobacco use among people with mental illness, which will ultimately recommend tailored interventions to curb the double-edged issue. Planning and implementing cessation advice through identified predictors and adhering to culturally specific interventions will provide outstanding results in clinical settings. Moreover, this review emphasizes the lack of smoking cessation advice from healthcare professionals when receiving mental illness treatment. This surge in the need for consistent screening by healthcare providers to support this vulnerable group emphasizes the need for integrated care, with tobacco cessation specialists in mental healthcare settings implementing tobacco control with the first visit to the healthcare provider. The findings firmly suggest strengthening tobacco control policies in Asia, especially in vulnerable groups, by making them inaccessible and unaffordable for use.

## Supplementary Information

Below is the link to the electronic supplementary material.

Supplementary file1 (DOCX 27 KB)
