# Supplementary material for: Correlates of Tobacco Use Among People with Mental Illness Within Asia: A Scoping Review
Source: Community Ment Health J. 2024 Aug 10;61(1):147–57. doi: 10.1007/s10597-024-01336-w (PMC11703933; doi:10.1007/s10597-024-01336-w)
Supplement: Supplementary file 1 — Supplementary file1 (DOCX 27 KB) [file 10597_2024_1336_MOESM1_ESM.docx]

**Supplementary** **Table 1: Prevalence and Predictors in selected studies**

| **Author (Year)** | **Country (Sample size)** | **Clinical Diagnosis (Setting)** | **Tobacco Screening tool** | **Current Prevalence (Measure of precision with 95% confidence)** | **Predictors** | **Limitations** |
| --- | --- | --- | --- | --- | --- | --- |
| Kelkar et al.  (2020) | India (100) | Schizophrenia (Hospital) | Not mentioned | 44%  (34.4% to 53.6%) | Male, older, low sociosconomic status,psychotic diagnosis (SCZ), family history, codependency and rural residence | - Small sample size - No standardised instrument - No biochemical test to confirm tobacco use |
| Khobragade et al.  (2020) | India (321) | SMI  (Hospital) | FTND & GATS | 12.14%  (9.04% to 15.24%) | Age, female, separated, undergraduate, nicotine dependence, psychotic diagnosis (BD), & no medical advice to quit | - Only OPD patients & no control group - Only participants from lower socioeconomic status - No assessment of the illness's clinical stage, active symptoms, and treatment factors. |
| Kar et al.  (2020) | India (303) | SMI  (Hospital) | Not mentioned | 40%  (34.1% to 45.9%) | Male, education up to primary school, economic independence, family history, psychiatric & medical comorbidities, and lower adherence to treatment. | - Recall biases (self-report by patients and their accompanying caregivers) - No confirmatory tests such as urine drug screening - No focus on the pattern of tobacco use (form of tobacco used, age of onset, duration, frequency, & amount used) |
| Srivastava et al.  (2018) | India (160) | SMI  (Hospital) | Not mentioned | 48.8%  (41.7% to 55.9%) | Male, lower education,unmarried, psychotic diagnosis, and codependency | - Small sample size & only OPD patients - No standardised instrument |
| Ranganathan et al. (2017) | India (75) | Schizophrenia (Hospital) | FTND | 25.3%  (15.1% to 35.5%) | Single, older, unemployed,rural residence, family history, the onset of smoking before mental illness, higher psychotropic drug dose, higher nicotine dependence, severe depression and anxiety scores and denial of smoking as an illness | - Small sample size - Observer Bias (Non-blinding of rater in smoking status) |
| Swaroop et al. (2014) | India (153) | Depression (Community) | FTND | 47.1%  (38.1% to 56.1%) | Low education, codependency & high nicotine dependence. | - Small Sample size & no male participants |
| Vatss et al.  (2012) | India (200) | Schizophrenia (Hospital) | FTND | 48% (41.3% to 54.7%) | Younger, single, unemployed, early initiation of tobacco, severe psychiatric symptoms, impaired global functioning and cognitive impairment. | - No female participants - Treatment status was not considered |
| Srinivasan & Thara et al. (2002) | India (687) | Schizophrenia (Hospital) | Self-report | 69.3%  (66.2% to 72.4%) | Single, psychotic diagnosis (SCZ), economic dependence & tension-relieving coping mechanism | - Self-reported method for tobacco use - No standardised instrument |
| Fang et al.  (2019) | China (469) | Schizophrenia (Hospital) | Self-report | 20.9%  (18.2% to 23.6%) | Male, family history of smoking, & severe psychiatric symptoms | - Self-reported method for tobacco use - No other smoking variables (Number of cigarettes per day, age of smoking onset, and levels of nicotine dependence). |
| Li et al.  (2017) | China (1102) | SMI-  (Hospital) | Self-report | 16.7%  (14.9% to 18.5%) | Male, low education, older, major-medical conditions, family history, psychotic diagnosis (SCZ &BP), age of onset, length of illness and frequent admission. | - Self-reported method - One tertiary hospital only - Lifestyle, social support, and nicotine dependence were not examined |
| Wang et al.  (2015) | China (100) | Psychiatric illness (Hospital) | FTND & KAS | 85%  (78.1% to 91.9%) | Male, older, low education, unemployed, psychotic diagnosis, family history, smoking as a stress coping mechanism, limited knowledge and awareness about cessation and minimal assessment by healthcare providers. | - Small sample size - Self-structured tool used |
| He et al.  (2014) | China (5996) | Depression  (Hospital) | FTND | 3.6%  (3.0% to 4.2%) | Young, single, low education, unemployed, family history, cultural influences, comorbidity, stressful experiences, & more protective parenting | - Only females - Hospital setting only - No biochemical test to validate self-reported smoking. |
| Zhang et al.  (2010) | China (1336) | Schizophrenia (Hospital) | FTND & CO | 79.7%  (78.2% to 81.8%) | Single, younger, early onset of illness, & psychotic diagnosis (SCZ). | - Recall Bias (self-reported age of onset) - Only young male participants - Hospitalised chronically ill patients only |
| Ma et al.  (2009) | China (1789) | SMI  (Community) | Self-report | 32.7%  (31.1% to 34.3%) | Single, male, low education (less than college), employed and psychotic diagnosis (MDD) | - DSM-III-R rather than DSM-IV |
| Zhang et al.  (2007) | China (130) | Schizophrenia (Hospital) | Self-report | 80%  (72.92% to 87.08%) | Severe psychotic symptoms | - Only male participants - Hospitalised chronically ill patients only - No standardised test |
| Washdev et al.  (2018) | Pakistan (276) | Schizophrenia (Hospital) | Not mentioned | 54%  (47.9% to 60.1%) | Males, single, chronic psychotic illness, & severe psychiatric symptoms | - No control group - No information regarding patients' socioeconomic status and education |
| Aziz et al.  (2017) | Pakistan (100) | Psychiatric illness  (Hospital) | CAGE | 45%  (35.4% to 54.6%) | Nicotine dependence, Anxiety or worry & boredom. | - Not clearly stated type of psychiatric illness. |
| Asharani et al.  (2020) | Singapore (380) | SMI –  (Hospital) | FTND & modified GATS | 39.5%  (35.3% to 44.7%) | Male, low education, psychotic diagnosis (MDD & SCZ), family history, as a coping mechanism (withdrawal symptoms & enjoy smoking), low-risk perception (physical health issues), nicotine dependence & low awareness of the institution's cessation programme | - Recall Bias (Self-report data collection: age of initiation, smoking patterns and types of services used) and convenience sampling. |
| Chong et al.  (1996) | Singapore (195) | Schizophrenia (Hospital) | Semi-structured interview | 31.8%  (26.1% to 37.9%) | Severe psychotic symptoms | - Sample not selected randomly |
| Shinozaki et al.  (2011) | Japan (70) | Schizophrenia (Hospital) | FTND & CO | 40.7%  (28.1% to 53.9) | Male, older, psychotic diagnosis (SCZ) | - Small sample size |
| Mansour et al.  (2017) | Jordan (203) | Schizophrenia, BPD & MDD - (Hospital) | DUSI-R | 67.5%  (62.0% to 74.0%) | Male, psychotic diagnosis (SCZ), & availability of cigarettes & co-dependency | - No frequency or severity of the psychotic symptoms - No assessment of health professionals' attitudes toward psychiatric illness |
| Kim et al.  (2013) | Korea (573) | Schizophrenia (Hospital) | FTND | 51.9%  (48.3% to 55.7%) | Males with no intention to quit and low quit advice from health professionals, nicotine dependence & co-dependency | - Hospital setting only - Only daily smokers assessed - Self-report survey |
| Yee et al.  (2012) | Malaysia (121) | Bipolar Disorders (Hospital) | FTND | 22.3%  (13.7% to 30.3%) | Male, low education, single, older, family history, psychotic illness, & frequent psychiatric hospitalisations | - Private hospital (pay for treatment) - No control group - Small sample in nicotine dependent group |
| Hapangama et al.  (2013) | Sri Lanka (325) | SMI  (Hospital) | DUS | 18.2%  (14.5% to 21.5%) | Male, younger, low education, psychotic diagnosis (SCZ), severe symptoms, smoking as a coping mechanism (to alleviate boredom, to improve attention) & co-dependency | - No use of biochemical tests - No rural participants |
| Liao et al.  (2002) | Taiwan (275) | Schizophrenia (Hospital) | Not report | 40.9%  (34.8% to 47.2%) | Male, older, psychotic diagnosis (SCZ), & severe positive symptoms. | - Chronic inpatients only |

***Legends*:** SLT- Smokeless Tobacco, SCZ-Schizophrenia,BD- Bipolar disoders, SMI-Severe Mental Illness, FTND- Fagerstrom Test for Nicotine Dependence, GATS-Global Adult Tobacco Survey, KAP-Knowledge, Attitudes and Services Questionnaire, CAGE- Cut, Annoyed, Guilty, and Eye Questionnaire, CO-Carbon monoxide, DUSI-R -Domain-I of Drug Use Screening Inventory, & DUS- Drug Use Scale, MMSE-Mini Mental Status Examination, PANSS-Positive and Negative Syndrome Scale, & BPRS-Brief Psychiatric Rating Scale.

**Supplementary Table 2: Predictors for tobacco use among people with mental illness.**

| **Predictor (n=studies reporting on this variable)** | | **Positive association** | **Negative association** |
| --- | --- | --- | --- |
| **Socio-demographic** | Advancing age (n=15) | Kelkar et al. (2020); Khobragade et al. (2020); Ranganathan et al. (2017); Li et al.(2017); Wang et al. (2015); Shinozaki et al. (2011); Yee et al. (2012); Liao et al. (2002) | Li et al. (2017); Zhang X. et al.(2010); Vatss et al. (2012); He et al.(2014); Zhang et al. (2010); Ma et al. (2009); Hapangama et al. (2013) |
|  | Gender: Male (n=18) | Kelkar et al. (2020); Kar et al. (2020); Srivastava et al. (2018); Fang et al. (2019); Li et al. (2017); Wang et al. (2015); Ma et al. (2009); Washdev et al. (2018)  Asharani et al. (2020); Shinozaki et al. (2011); Mansour et al. (2017); Kim et al. (2013); Yee et al. (2012); Hapangama et al. (2013); Liao et al. (2002) | Khobragade et al. (2020); Swaroop et al. (2014); He et al. (2014) |
|  | Education: Lower (n=11) | Khobragade et al. (2020); Kar et al. (2020); Srivastava et al. (2018); Swaroop et al. (2014); Li et al.(2017); Wang et al. (2015); He et al.(2014); Ma et al. (2009); Asharani et al. (2020); Yee et al. (2012); Hapangama et al. (2013) |  |
|  | Marital status: Married (n=11) | Khobragade et al. (2020); Li et al.(2017) | Srivastava et al. (2018); Vatss et al. (2012); Srinivasan & Thara et al. (2002); Wang et al. (2015); He et al. (2014); Zhang et al. (2010); Ma et al. (2009); Washdev et al. (2018); Yee et al. (2012) |
|  | Family history: tobacco (n=10) | Kelkar et al. (2020); Kar et al. (2020); Ranganathan et al. (2017); Vatss et al. (2012); Fang et al. (2019); Li et al.(2017); Wang et al. (2015); He et al. (2014); Asharani et al. (2020); Yee et al. (2012) |  |
|  | Employment: No (n=7) | Vatss et al. (2012); Li et al. (2017); Wang et al. (2015); He et al. (2014) | Kar et al. (2020); Ranganathan et al. (2017); Ma et al. (2009) |
|  | Socio economic status: Lower (n=2) | Kelkar et al. (2020) | Srinivasan & Thara et al. (2002) |
|  | Onset of smoking before mental illness (n= 5) | Vatss et al. (2012); Ranganathan et al. (2017); Li et al.(2017); He et al.(2014); Zhang et al. (2010) |  |
|  | Residence: Rural (n= 2) | Kelkar et al. (2020); Ranganathan et al. (2017) |  |
| **Clinical variables** | To relieve psychiatric symptoms (n=4) | Fang et al. (2019); Zhang et al. (2007); Washdev et al. (2018) | Ranganathan et al. (2017) |
|  | Psychotic diagnosis (n=23) | Kelkar et al. (2020); Khobragade et al. (2020); Kar et al. (2020); Srivastava et al. (2018); Ranganathan et al. (2017); Vatss et al. (2012);Srinivasan & Thara et al. (2002); Fang et al. (2019); Li et al.(2017); Wang et al. (2015); He et al. (2014); Zhang et al. (2010); Ma et al. (2009); Zhang et al. (2007); Washdev et al. (2018); Asharani et al. (2020); Chong et al. (1996); Shinozaki et al. (2011); Mansour et al. (2017); Yee et al. (2012); Hapangama et al. (2013); Liao et al. (2002) | Swaroop et al. (2014) |
|  | Co-morbidity: Physical condition (n=3) | Kar et al. (2020); Li et al.(2017); He et al.(2014) |  |
|  | Nicotine dependence (n=8) | Khobragade et al. (2020); Ranganathan et al. (2017); Swaroop et al. (2014); Vatss et al. (2012); He et al. (2014); Aziz et al. (2017); Asharani et al. (2020); Kim et al. (2013) |  |
|  | Co-dependency (n=8) | Kelkar et al. (2020); Khobragade et al. (2020); Kar et al. (2020); Srivastava et al. (2018); Ranganathan et al. (2017); Swaroop et al. (2014); Mansour et al. (2017); Kim et al. (2013) |  |
| **Individual variables** | Lack of knowledge (n=7) | Kar et al. (2020); Wang et al. (2015); Asharani et al. (2020); Ranganathan et al. (2017); Khobragade et al. (2020); Srinivasan & Thara et al. (2002); Kim et al. (2013) |  |
|  | Lack of motivation (n=9) | Srinivasan & Thara et al. (2002); Asharani et al. (2020); Kim et al. (2013); Li et al.(2017); Fang et al. (2019); Wang et al. (2015); Srinivasan & Thara et al. (2002); Asharani et al. (2020); Aziz et al. (2017) |  |
|  | Coping mechanism (n=6) | Ranganathan et al. (2017); Srinivasan & Thara et al. (2002); Aziz et al.(2017); Asharani et al. (2020); Hapangama et al. (2013); He et al.(2014) |  |

**Supplementary** **Table 3: Quality assessment checklist: CASP Tool**

| **Author** | **Year** | **Purpose** | **Methodology** | **Research design** | **Recruitment sampling** | **Data collection** | **Ethics** | **Data analysis** | **Findings** | **Value of research** | **Overall score** |
| --- | --- | --- | --- | --- | --- | --- | --- | --- | --- | --- | --- |
| Asharani, P. et al. | 2020 | √ | √ | √ | √ | √ | √ | √ | √ | √ | A |
| Aziz, R. et al. | 2017 | √ | √ | √ | √ | √ | X | * | √ | * | C |
| Chong, S. et al. | 1996 | √ | * | √ | * | √ | X | √ | √ | * | C |
| Fang, Y. et al. | 2019 | √ | √ | √ | √ | √ | √ | √ | √ | √ | A |
| Hapangama, A. et al. | 2013 | √ | √ | √ | √ | √ | √ | √ | √ | √ | A |
| He, Q. et al. | 2014 | √ | √ | √ | √ | √ | √ | √ | √ | √ | A |
| Kar, S. et al. | 2020 | √ | √ | √ | √ | √ | √ | √ | √ | √ | A |
| Zhang, X. et al. | 2007 | √ | √ | √ | √ | √ | √ | √ | √ | √ | A |
| Zhang, X. et al. | 2010 | √ | √ | √ | √ | √ | √ | √ | √ | √ | A |
| Yee, A. et al. | 2012 | √ | √ | √ | √ | √ | √ | √ | √ | √ | A |
| Washdev et al. | 2018 | √ | √ | * | √ | √ | * | √ | √ | √ | C |
| Wang, X. et al. | 2015 | √ | √ | √ | √ | √ | √ | √ | √ | √ | A |
| Vatss, S. et al. | 2012 | √ | √ | √ | √ | √ | √ | √ | √ | √ | A |
| Swaroop, N. et al. | 2014 | √ | √ | √ | √ | √ | √ | √ | √ | √ | A |
| Srivastava, R. et al. | 2018 | √ | √ | √ | √ | √ | √ | √ | √ | √ | A |
| Srinivasan, T. et al. | 2002 | √ | √ | √ | √ | √ | √ | √ | √ | √ | A |
| Shinozaki, Y. et al. | 2011 | √ | √ | √ | √ | √ | √ | √ | √ | √ | A |
| Ranganathan, T. et al. | 2017 | √ | √ | √ | √ | √ | √ | √ | √ | √ | A |
| Ma Xin, X. et al. | 2009 | √ | √ | √ | √ | √ | √ | √ | √ | √ | A |
| Liao, D. et al. | 2002 | √ | √ | √ | * | √ | √ | √ | √ | √ | B |
| Kim, S. et al. | 2013 | √ | √ | √ | √ | √ | √ | √ | √ | √ | A |
| Kelkar, P. et al. | 2020 | √ | √ | √ | * | √ | √ | √ | √ | √ | B |
| Mansour, A. et al. | 2017 | √ | √ | √ | √ | √ | √ | √ | √ | √ | A |
| Li, X. et al. | 2017 | √ | √ | √ | √ | √ | √ | √ | √ | √ | A |
| Khobragade, B. et al. | 2020 | √ | √ | √ | √ | √ | √ | √ | √ | √ | A |

**Legends:** Key to ratings: ✓ = Detailed coverage of screening question; * = Screening question covered but not detailed; X= Screening question not addressed. Overall quality rating: A= Nil or few flaws, the study credibility, transferability, dependability, and confirmability is high; B= some flaws, unlikely to affect the credibility, transferability, dependability, and/or confirmability of the study; C = some flaws which may affect the credibility, transferability, and/or confirmability of the study; D = significant flaws which are very likely to affect the credibility, transferability, dependability and/or confirmability of the study(CASP 2017)**.**
